# Three Dimensional UAV Positioning for Dynamic UAV-to-Car Communications

**DOI:** 10.3390/s20020356

**Published:** 2020-01-08

**Authors:** Seilendria A. Hadiwardoyo, Carlos T. Calafate, Juan-Carlos Cano, Kirill Krinkin, Dmitry Klionskiy, Enrique Hernández-Orallo, Pietro Manzoni

**Affiliations:** 1 Department of Electronics ICT—IDLab, Universiteit Antwerpen—imec, 2000 Antwerp, Belgium; 2 Department of Computer Engineering (DISCA), Universitat Politècnica de València, 46022 Valencia, Spain; ehernandez@disca.upv.es (E.H.-O.); pmanzoni@disca.upv.es (P.M.); 3 Department of Software Engineering & Computer Applications (MOEVM), St. Petersburg Electrotechnical University “LETI”, 197022 St. Petersburg, Russia; kvkrinkin@etu.ru (K.K.); dmklionsky@etu.ru (D.K.)

**Keywords:** PSO, genetic algorithm, ITS, UAV, simulation, dynamic positioning, 3D placement, vehicular communications

## Abstract

In areas with limited infrastructure, Unmanned Aerial Vehicles (UAVs) can come in handy as relays for car-to-car communications. Since UAVs are able to fully explore a three-dimensional environment while flying, communications that involve them can be affected by the irregularity of the terrains, that in turn can cause path loss by acting as obstacles. Accounting for this phenomenon, we propose a UAV positioning technique that relies on optimization algorithms to improve the support for vehicular communications. Simulation results show that the best position of the UAV can be timely determined considering the dynamic movement of the cars. Our technique takes into account the current flight altitude, the position of the cars on the ground, and the existing flight restrictions.

## 1. Introduction

UAVs can be easily deployed as information relays for emergency scenarios thanks to their flexibility. Since they can be positioned at high altitudes compared to ground infrastructure deployments, UAVs can achieve long-range signal transmissions with better Line-of-Sight (LOS) conditions. Hence, compared to standard ground infrastructure relays, UAVs can offer significant advantages [1]. For instance, UAVs can become mobile infrastructure elements in situations where the existing infrastructures are limited, as in rural areas or even in urban areas when emergency situations take place. Other uses of UAVs include improving Intelligent Transportation Systems (ITS) on disaster assistance operations [2] and performing remote sensing [3], among others.

In terms of supporting vehicular communications, UAVs can relay information for vehicles on the ground when a direct multihop link between them is not available [4]. A UAV can move freely in three-dimensional space without having to follow routes or specific trajectories, a feature that ground vehicles do not have. Also, a UAV can be deployed as a mobile Road Side Unit (RSU) since it flies above high buildings in urban areas that can obstruct the communication between cars on the ground [5]. This can be the solution to providing continuous connectivity among the cars. The presence of UAVs as relays for vehicular communication can assist in forwarding messages when direct car-to-car communication is not possible. Differently from cars, in which the movement is limited to the two dimensional space and to specific roads, UAVs can become an alternative to provide connectivity since they have no space constraints [6].

In three dimensional scenarios that involve UAVs, the diffraction caused by buildings, mountains, or high-level terrains blocking the signal is likely to occur [6]. Placing the UAV at a high altitude can be a solution to avoid the Non-Line-of-Sight (NLOS) conditions caused by high-level terrains such as hills or mountains. Nonetheless, the communication range decreases if the UAV is placed too high, and in addition, there are regulations that restrict the permitted flight height of the UAV. Hence, the position of the UAV has to be determined in the 3D environment, where it can still maintain the signal range towards the receiver by adapting its position to avoid NLOS conditions, while still respecting the maximum flight height allowed, among other flight restrictions. Thus, to account for various dynamic constraints, a solution able to determine the optimal location throughout the time should be found [7].

In this paper, a dynamic positioning technique for UAVs is proposed by implementing optimization algorithms to find the best position of a UAV in a dynamic vehicular communications environment. The position adopted by the UAV at any instant of time will allow it to support the communication between cars on the ground. Such an optimum position is obtained by considering the signal quality received by the cars acting as signal receivers.

We have used a previously developed model [8] to calculate the signal quality. The model considered the 3D environment with terrain irregularities that might obstruct the communications. The goal is having the UAV as a relay placed in its optimum position at each specific time instant so that it can forward information from one car to another when direct car-to-car communications are obstructed by high terrains in the 3D space. In this research work, we have used a Particle Swarm Optimization (PSO) algorithm and a genetic algorithm (GA) that helped us to find the best position of the UAV throughout the experiment. The ideal and desired case is that the UAV can still achieve adequate signal conditions towards every car on the ground [9]. The results show that the positioning technique can find the optimized position by defining its best altitude adequately that it is not hindered by terrain blockages.

The paper is organized as follows: the research works related to our proposal are discussed in the following section. In Section 3, we highlight the problems to be tackled and our contribution in this paper, where we start by a discussion of the problem and the optimization algorithms used. In Section 4, the simulation framework which we have used to evaluate our algorithm is presented, as well as its setup. Afterwards, in Section 5, we present the results and discuss the main findings. Finally, in Section 6, we present the conclusions and discuss future works.

## 2. Related Works

Several research efforts have been documented involving UAVs as nodes deployed to provide connectivity. Lin et al. [10] studied the deployment of LTE connectivity for UAVs. These authors highlighted some challenges such as LOS propagation in the sky. Other related research work was performed by Van der Bergh et al. [11], where they present an analysis of the impact of LTE-enabled UAVs on an already existing LTE ground network. The authors studied the case of having UAVs as user equipment and base stations. The work of Nguyen et al. [12] highlighted the interference that might occur between terrestrial and aerial-based radio connectivity.

Using simulation, researchers have investigated the methods to optimize the communications between UAVs and cars. One of the research efforts done was analyzing the deployment of lesser amount of UAVs for communications by having an optimal altitude [13]. In [14], the authors analyze the characteristics of communications between drone and vehicle in terms of delay. When supporting connectivity for the groups of cars that are disconnected, drones can link these groups as relays [15]. Nevertheless, in order to get the optimum connectivity between the UAVs and cars, it is needed to find the best location to place the UAVs so that it can transmit adequate signals to the ground vehicles.

Node placement is crucial when it comes to wireless networks where the nodes are located freely in space. To get the best performance in wireless networks, placement strategies can be useful [16]. An algorithm for base station placement to maximize the network capacity was proposed in [17]. Another proposal that instead seeks to maximize the network lifetime is [18].

In car-to-car communications, the placement of nodes that act as relays affects the performance of information dissemination. Optimal placement of RSUs is discussed in [19] with the aim of improving connectivity at intersections. Looking into the number of vehicle reports in the communication range of the RSUs, the proposed scheme can find the best location for these RSUs. Other works [20] proposed a method to determine the RSU deployment so that it is able to maximize the number of vehicles within radio range. Anther placement method was proposed in [21], being able to minimize the average report time from cars to RSUs.

The idea of having UAVs as mobile infrastructure units has been investigated by various researchers. Chiaraviglio et al. [22] investigated the use of small cells on top of the UAVs to cover hotspot areas. Huang et al. [23] proposed a novel coordinated path planning algorithm for multi-UAVs to deal with the trajectory smoothing problem using optimization algorithms. The work in Reina et al. [24] focused on the application of a metaheuristic algorithm to solve multi-objective coverage problems of UAV networks. The issue with battery fuel capacity of the UAV was analyzed by Song et al. [25]. Another idea to overcome the energy issue is proposed by Trotta et al. [26]. In their work, the UAVs that monitor a set of points of interest make use of the public bus network for recharging. This is solved by Mixed Integer Linear Programming techniques, where the formulation identifies the UAVs, the next bus, and the next point of interest.

Specifically talking about UAV placement in 3D environments, proposals such as [27] aim at determining the best place for a UAV to provide connectivity for nodes in indoor buildings affected by a disaster. Nodes, which are the users inside the building, can be covered by a strategically located UAV so that the total transmitted power is kept to a minimum. Another work [9] proposes a method to position a team of UAVs so as to maximize the user coverage ratio in a 5G network. Determining the optimal placement for a drone can be done using a PSO algorithm as well, as discussed in [28], where a coverage area can be maximized while still considering the drone capacity in the scope of public safety and disaster management. Considering the lifetime of drones to maximize the total throughput of the receivers was also discussed in [29] when deploying and positioning a swarm of drones.

A topic covered in [30] was deploying UAVs as aerial base stations in a three-dimensional environment. The proposed work was about deploying UAVs to support cellular networks as it allows extending the coverage area. In another similar work [31], UAVs are deployed to support cellular networks in the presence of dynamic events. When deploying UAVs that support vehicular communications, a UAV position can be determined using an algorithm proposed by [32], so that it offers Quality of Service (QoS) communications to the cars on the ground. In our work, we provide a technique to support the communication of the moving nodes on the ground, in which in this case the nodes are the cars.

In this work, we seek to determine the optimal position of a UAV involved in UAV-to-car communications. As the cars on the ground are moving dynamically, the UAV position should be adjusted throughout the time so that it adapts to the radio links towards the different cars involved. In our work, we used optimization algorithms such as Particle Swarm Optimization (PSO) and Genetic Algorithm (GA) to determine the optimum position of the UAV in terms of achieving the best coverage for the cars on the ground at each specific time. Since the environment is three dimensional, the altitudes of the cars and of the UAV are taken into account at all times. In addition, the irregularities of the terrains are also considered when determining the UAV location and the channel quality towards the different ground vehicles. Hence, the optimization algorithm is used for finding the best position of a UAV in an area where the connectivity is minimum due to the lack of infrastructure. In such scenarios, the connection between cars cannot be maintained since the Line of Sight in the mountainous area is hindered by mountains. Thus, the whole three dimensional space (above ground) is considered as feasible for the UAV to explore with the aim of offering connectivity to the mobile targets on the ground.

## 3. Optimum UAV Positioning

In this section, we describe the formulation of the positioning problem. In particular, we will analyze how the optimization algorithm solves the positioning problem of nodes. We will also explain in detail the two optimization algorithms used in this research work, which are the Particle Swarm Optimization (PSO) algorithm and a genetic algorithm (GA). In addition, by considering a 3D environment, irregularities of the terrains might affect the signal transmitted; hence, we will also discuss in more detail the path loss model taken into account when proposing the different UAV positioning strategies.

### 3.1. Problem Formulation

For our study, a rural area scenario is chosen since it has hilly roads and significant terrain irregularities. This way, we can test whether the UAV position in 3D is affected by the terrain levels. Notice that terrain irregularities often cause that, when two cars are communicating, there might be a high chance that there is a hill in between. Thus, the cars experience NLOS conditions in their radio link due to the presence of hills, which make necessary the presence of information relays to maintain the connectivity between these cars. Considering that most rural areas lack enough infrastructure so as to help in information relaying in such cases, alternatives must be sought.

**Figure 1:**
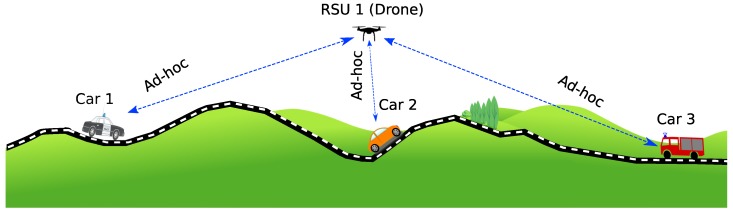
Unmanned Aerial Vehicle (UAV) acting as a Mobile Road Side Unit (RSU) [33].

As depicted in Figure 1, cars on the ground might not have the possibility to connect directly with each other, or they can experience very poor link conditions due to the NLOS features of the wireless channel caused by hills. One solution to provide the necessary connectivity among them is to deploy a UAV that can act as a mobile relay. This way, the UAV can forward information from one car to another. In our work, the experiments are made in a simulated environment where the cars follow a specific trajectory on an existing road. Regarding the UAV location, it should change throughout time so as to adjust to the locations of the different cars involved, which are updated every second.

The position of the UAV is adjusted depending on the signal strength received by the cars on the ground. A new optimal position has to be found at every time step as cars are moving. In particular, the new position should allow the UAV to maintain the best possible communications link with the cars by considering signal levels. As the sender, or the UAV in this case, transmits the signal in a three-dimensional environment to the receivers (the different cars in our case), possible NLOS conditions must be accounted for in the end-to-end link. Hence, the UAV is positioned at a certain altitude in a way that it can achieve good visibility levels towards the current location of a target car.

### 3.2. Optimization Problem

To determine whether our positioning technique is optimal or not, the metric used is the Received Signal Strength Indicator (RSSI) value measured at the different receivers (cars). So, the optimal UAV location is defined based on the average RSSI value. In particular, the average RSSI should be greater than a minimum threshold of -89 dBm to achieve full coverage connectivity. This value is the main criteria to determine that the placement meets the desired conditions. In detail, we analyze the RSSI at each car when the UAV’s position is selected. The RSSI is calculated through a network simulator. Both the position of the UAV and the car are inserted as inputs to the simulator. This repeats when a candidate position of the UAV is to be tested, and when the position of the car changes.

To calculate the RSSI, the path loss model is taken into account depending on the location of the nodes. This way we can determine whether an obstacle might be present and blocking the communication between two nodes. In other words, this condition is called knife-edge diffraction, where the blocking obstacle is the knife. The main objective in our work is to find the best location of the UAV as a sender in a three-dimensional environment in such a way that the signals transmitted by the UAV can be received by the cars on the ground with adequate reception power.

A simple but inefficient solution would be doing an exhaustive search. By doing this, every location in the search space should be explored and tested so that the best definitive location can be found at every time step. Nevertheless, doing an exhaustive search can take a lot of time and consume significant resources. A more sophisticated yet effective solution would be adopting a meta-heuristic optimization algorithm. Using this strategy, optimum values could be obtained without having to test every possibility in the search space. Possibly, random values of candidate locations would be defined at the beginning, but the algorithm would search the other best possible candidates in an iterative manner. In the case of finding the UAV position, to get the most precise optimum position we can do an exhaustive search. However, since the possibilities to explore are too many with respect to the values of longitude, latitude, and altitude as inputs, the search may take forever. Hence, to offer quicker solution in finding a position in 3D area, the optimization techniques can be performed. Thus, based on two different meta-heuristic optimization techniques, two algorithms will be implemented in our positioning technique, which will be covered in more detail in the following subsection.

### 3.3. Particle Swarm Optimization

An optimization algorithm inspired by the social behavior of animals, such as flocks of birds an schools of fish, is the Particle Swarm Optimization (PSO) [34] algorithm. As part of swarm intelligence, the algorithm improves a candidate solution, or particle, through iterations starting from random solutions, and picking the best experience of each iteration (BestLoc), and the best global experience from all the iterations (GlobalBest). A particle (Loc), or a candidate location, is a possible position for our UAV (pos). In each iteration (t+1), the best location of each particle ($loc_{i}$) and the best global location are updated. The main parameter affecting the updates is the velocity (*V*). The velocity in PSO is a distance achieved by a particle, or a candidate following location, from its current or previous location at an iteration. The velocity is affected by the inertial weight (*W*) when it is varied (in most cases it has a value of 1), the acceleration coefficients (c1 and c2, which both have the value of 2), and the random numbers (r1 and r2) uniformly distributed in the interval between 0 and 1. At the beginning, the algorithm has a population of candidate solutions. The equation to calculate the velocity that defines the updates is:

(1) $$\begin{array}{c} V_{i}\left(t+1\right)=W\times V_{i}\left(t\right)+r1\cdot c1\cdot \left(BestLoc_{i}\left(t\right)-Loc_{i}\left(t\right)\right)+\\ r2\cdot c2\cdot (GlobalBest\left(t\right)-Loc_{i}\left(t\right)) \end{array}$$

The following or next location of the particle is obtained by adjusting the velocity to the current or previous location of the particle as:

(2) $$\begin{array}{c} Loc_{i}\left(t+1\right)=Loc_{i}\left(t\right)+V_{i}\left(t+1\right) \end{array}$$

Algorithm 1 shows the implementation of PSO algorithm. The algorithm begins by having a population with random locations. Hence, a set of particles representing candidate locations makes up our population. The candidate locations, which are obtained randomly, are limited by the variables set as maximum ($var_{min}$) and minimum ($var_{min}$). With the initial velocity, which is zero at the beginning and initial locations, the function will find the cost and calculate the initial value, which in our case is the RSSI (rssi) value for each particle. The best RSSI of all particles (Bestrssi) will be defined as the global best. PSO will then find a new value after having the initial position and RSSI for all the particles. The new value is obtained by calculating the new velocity based on the local best position (Bestpos), and the global best position (GlobalBestpos). The value of each position will be updated according to the new velocity value, which in turn can return a new GobalBestPos. The Bestpos is updated if the new position is better. The same happens to the GlobalBestpos if its new value after the iteration is better than the one from the previous iteration. By doing more iterations with newly calculated velocities, we can get refine the solution until the best value for the rssi is found.
**Algorithm 1** Particle Swarm Optimization (PSO) Algorithm.**Input:**        Maximum number of iterations (MaxIt).        Population size (PopSize).        Lower and upper bound variables ($var_{min},var_{max}$).**Output:**        Best value of all particles (GlobalBestrssi).1:**for** i=1:PopSize **do**2:    $V_{i}=0$3:    $pos_{i}=rand\left(var_{min},var_{max}\right)$4:    $rssi_{i}=pathloss\left(pos_{i}\right)$5:    $Bestpos_{i}=pos_{i}$6:    **if** $Bestrssi_{i}<GlobalBestrssi$ **then**7:        $GlobalBestrssi=Bestrssi_{i}$8:    **end if**9:**end for**10:**for** t=1:MaxIt **do**11:    **for** i=1:PopSize **do**12:        $V_{i}\left(t\right)=W*V_{i}\left(t\right)+r1*c1*\left(Bestpos_{i}\left(t\right)-Loc_{i}\left(t\right)\right)+r2*c2*\left(GlobalBestpos\left(t\right)-pos_{i}\left(t\right)\right)$13:        $pos_{i}\left(t\right)=pos_{i}\left(t\right)+V_{i}\left(t\right))$14:        $rssi_{i}=pathloss\left(pos_{i}\right)$15:        **if** $rssi_{i}<Bestrssi_{i}$ **then**16:           $Bestpos_{i}\left(t\right)=pos_{i}\left(t\right)$17:           $Bestrssi_{i}=rssi_{i}$18:           **if** Bestrssi<GlobalBestrssi **then**19:               $GlobalBestrssi=Bestrssi_{i}$20:           **end if**21:        **end if**22:    **end for**23:**end for**

### 3.4. Genetic Algorithm

A Genetic Algorithm (GA) is a non-deterministic optimization method based on genetic theory [35]. GA simulates the evolution of a population of candidate solutions to optimize a problem. The population or candidate solution adapts to the environment over the generations, being these generations renewed through iterations. This iterative process resembles biological behaviors like the crossovers of chromosomes, mutations of genes, and inversions of genes, processes that occur to living organisms over generations. In our work, we use a GA to simulate the evolution of a population of UAV locations adapting to the cost function. The cost function will be the same as the one in PSO: the average RSSI towards the different receivers.

As depicted in Algorithm 2, we first define that the population is a candidate set of optimum locations for the UAV acting as a relay for cars. The candidate set of optimum locations, defined as latitude, longitude, and altitude, is limited by the lower and upper bound variables ($var_{min},var_{max}$). At first, the populations are generated randomly, but having specific genes or characteristics. The genes, in this case are the latitude, longitude, and altitude associated with the UAV’s location. From those characteristics, we define the fitness, which in this case is the RSSI. Afterwards, we build a new generation. This is done by selecting the parents or the chromosomes in the current generation. The parents are chosen by randomly selecting two sets of chromosomes. After selecting the chromosomes, the genes inside the chromosomes are crossed over to create new chromosomes. This is when the crossover process occurs, consisting of combining the genes, or, in the case of this work, the location parameters (latitude, longitude, and altitude). With the new chromosomes, we then do a mutation. The mutation is performed to maintain the genetic diversity, or, in this case, to increase the number of candidate locations. With the mutated chromosomes, we determine the fitness, which in this case is the RSSI. After getting a new generation, this process is repeated according to the limit of the generations. With more and more generations produced, better RSSI values can be obtained.
**Algorithm 2** Genetic Algorithm (GA)**Input:**        Maximum number of generations (MaxGen).        Population size (PopSize).        Lower and upper bound variables ($var_{min},var_{max}$).**Output:**        Best value of all chromosomes (GlobalBestrssi).1:**for** i=1:PopSize **do**2:    $pos_{i}=rand\left(var_{min},var_{max}\right)$3:    $rssi_{i}=pathloss\left(pos_{i}\right)$4:    $Bestpos_{i}=pos_{i}$5:**end for**6:**for** t=1:MaxGen **do**7:    **for** i=1:PopSize **do**8:        SelectParents9:        Crossover10:        Mutation11:        $pos_{i}=pos_{i+1}$12:        $Bestpos_{i}=pos_{i}$13:        $Bestrssi_{i}=rssi_{i}$14:        $GlobalBestrssi=Bestrssi_{i}$15:    **end for**16:**end for**

### 3.5. Path Loss Model

**Figure 2:**
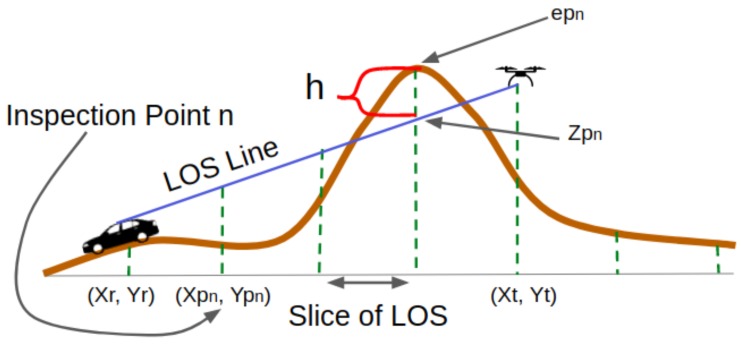
Detecting the Hills as Obstacles [8].

The RSSI value can be obtained through the path loss model. To derive the path model used in our work, we relied on our previous work [8]. With this model, the signal loss can be calculated by considering terrain features that act as obstacles. By considering the elevation information retrieved from a Digital Elevation Model (DEM), we can determine when the terrain affects the LOS between a sender and a receiver. A knife-edge is detected when there is a blocking terrain, as depicted in Figure 2. Through multiple knife-edge diffraction effects, the actual end-to-end loss is obtained. On our previous work [8], we opted for the Bullington model [36] as it offers a good trade-off between performance and computational costs.

According to the information obtained from the DEM, the path loss or signal attenuation can be calculated. By taking into account the elevation level of the terrain, we can get the height of the knife through the difference of altitude between the UAV and the car. Notice that, in rural areas, signal interferences from external sources are minimal, meaning that the terrain becomes the main factor affecting signal quality.

A DEM provides real-world terrain data that, for the purpose of our current work, can provide information about the elevation of the terrains with respect to the sea level. Since the terrains have different levels of elevation in the area, we can easily find whether the terrain is hilly, mountainous, or flat. This elevation information is obtained by indicating the latitude and the longitude of each selected location.

A LOS segment connecting the sender and the receiver can help us to spot the knife or the blocking terrains. The height of this segment and the terrain elevation in the locations along the segment are compared to determine whether the LOS is blocked by the terrain or not. An obstacle is present whenever the terrain level or the elevation is higher than the LOS segment height. This way, the signal loss can be calculated as the diffraction effect occurs. The signal attenuation is obtained by accounting for the height of the obstacle, the wavelength, and the distance between the obstacle and the sender and receiver terminals. In this case, the Fresnel-Kirchoff diffraction parameter will also define the signal attenuation. In our simulation framework, this path loss model is incorporated. This will be explained in more detail in Section 4.

## 4. Overview of Simulation Tools Used

In this section, we provide the details on how our proposed solutions are implemented in the simulation tools adopted. A testing framework was developed to get the best position of the UAV in a simulated environment. In addition, the simulated environment setup is also covered in this section.

### 4.1. Testing Framework

An application that runs the simulation was developed as a testing framework, which will implement both the PSO and the GA algorithms to determine the optimum placement of the UAV. This framework integrates OMNeT++ [37] as a network simulation tool, SUMO [38] as a vehicular mobility simulation tool to characterize the movement of the cars on the ground, as well as Veins [39] as a vehicular network simulation tool. Our application determines the location by having the average value of the signal received by the cars (RSSI) as the algorithm’s cost function value. This is obtained by executing the simulation tools and extracting the results.

**Figure 3:**
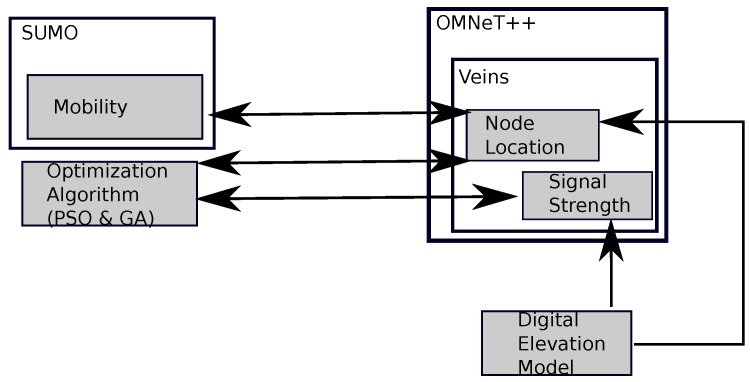
Testing Framework Architecture.

Figure 3 shows the architecture of our testing framework. The algorithm was implemented in a separate developed application as a framework. The cost function value or the best fitness of the algorithm was obtained by running the simulation tools combined (OMNeT++, SUMO, and Veins). The variables that affect the RSSI values obtained are the receivers’ locations as returned by SUMO. To detect the signal blockages or knife-edge effects that affect the signal strength, a path loss model in [8] was implemented.

By default, the parameters are selected when running the PSO and GA. For the PSO, the number of particles was set to 50, and the number of iterations was also set to 50, whereas in GA both the number of populations and the number of generations were set to 50. The exploration space for both algorithms is limited by the map space in the simulation. Both PSO and GA algorithms will have 50 candidate locations at first, before iterating or going through generations. By considering the cars’ locations determined by SUMO, Veins can determine the best RSSI value by testing every possible location.

In this particular case, the position of the UAV dynamically changes depending on the mobility of the cars on the ground. Default values of the commonly used parameters are chosen by using either PSO or GA. As a side note, these parameters can affect the optimality of the position. Having more iterations on the algorithm used can effectively result in a more optimum position. However, we should not neglect the fact that, in real deployments, more iteration times might result in having the cars’ position to change, making the results less effective. Since we are working with simulation time, and not real time, for practical purposes we consider the calculation involved to be instantaneous. Hence, the main goal is to determine the best-case performance achieved through the sequence of positions determined by PSO and GA.

The performance of finding the best UAV position depending on the cars’ position on the ground differs from time to time, and subject to the positions of the cars on the ground. Our main focus on this work is making a comparison of both algorithms while having similar parameters. Due to this fact, we did not highlight the matter of selecting the best parameters for algorithmic convergence. As a side note, making these algorithms converge can be a challenge since both PSO and GA are heuristics where no exact configuration or parameter choice guarantees obtaining the optimal result in a search space. Our maximum recorded time to get the optimum solution was with 320 particles, and the minimum recorded time was with 16 particles. Concerning GA, the maximum recorded time in getting the solution was with 760 generations, and the minimum recorded time was with 32 generations. The primary goal of this work is uniformly using the commonly used parameters in both PSO and GA, and then compare which one is more accurate and efficient.

### 4.2. Simulation Setup

We have defined a scenario to test the optimization algorithms. Specifically, we have chosen a mountainous rural area in Pont de Suert, Catalonia, Spain, near the Pyrenees Mountains. A real map of Pont the Suert was imported from Open Street Map (OSM) [40] to make the simulation more real. To complement the environment imported into the simulation, we have also considered the elevation information, which allowed us to have a complete characterization of the levels of the terrain in the chosen area. In particular, we have obtained it from the SRTM DEM [41]. One of the reasons why we have chosen this particular location is because, in this area, irregular terrains exist that are prone to hinder communications. The area imported has a size of 5000×5000 m in Cartesian coordinates. With this in mind, the exploration space for the optimization algorithm is defined. As for the altitude, it is limited to 120 m above the ground, which is the maximum flight height permitted in Spain.

**Figure 4:**
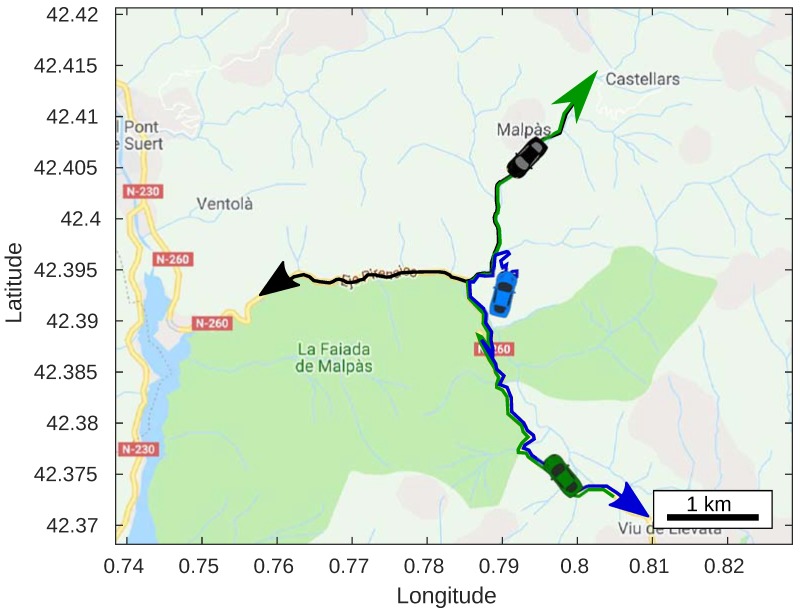
Trajectories for ground vehicles in our experiments.

In the scenario, we have placed three cars that have their trajectories defined according to Figure 4. In theory, the UAV that acts as a relay can offer the connectivity between two nodes or cars. However, we have considered three cars in the scenario to show that the UAV can simultaneously act as a data relay for more than two nodes. However, the number of cars served by the UAV should be taken into consideration carefully, since a growing number of cars will complicate the UAV positioning strategy.

As for simulating the UAV, which is of the hexacopter type, in the scenario, we assume that it has no limitations in terms of energy. The UAV is deployed on-demand at a specific time to act as a mobile relay. In addition, we have a single UAV in the scenario since our main focus is to find the optimal position for this UAV, and not on how multiple UAVs could be deployed to provide coverage, an alternative that is outside the scope of this work. It is assumed that the UAV carries a small embedded system having some computational power, performing better than e.g., a Raspberry Pi, in addition to an IEEE 802.11p wireless interface for communication with the vehicles, and a 4G interface for remote monitoring tasks. Overall, the payload weight remains below than 1 kg.

The position of the UAV is calculated by considering the current location of each car every second. The car positions are obtained by the UAV from their beacons. This way, we can assess whether the UAV offers optimal coverage when communicating with the cars. The SUMO traffic simulator generates the cars’ movements, and it simulates the scenario for 280 s. This limited time is due to the fact that, after that mentioned time limit, the cars will no longer be in the exploration space boundaries, in which the cars will be too far to each other that the communication will surely be out of range.

The UAV generates UDP packets in the scenario and broadcasts them to the cars on the ground. The mode of communication is ad-hoc in this case. The packets that are transmitted by the UAV are Basic Safety Messages (BSMs). Each second, the UAV sends 10 packets. In this scenario, an 802.11p connection is considered, with broadcast communications taking place. Table 1 details the parameters that are set in our simulation experiments.

## 5. Results

As explained in the previous section, our proposed solution to find optimal UAV positions was developed using simulation tools. After executing the test framework that runs the simulation, we have obtained the optimum sequence of UAV positions, as well as other useful information, such as the RSSI, time, and the position of the other nodes.

By using two different optimization algorithms, we have defined the optimal positions and, in turn, can derive the trajectory. Other results include the impact on altitude, received signal strength, total path length, and speed.

### 5.1. Uav Positions

We have obtained the results concerning the best locations of the UAV through the simulation experiments. The location information consists of latitude, longitude, and altitude. In addition, the value for the RSSI is also obtained for each UAV location at every second of the simulation. Thus, the locations of the UAV in a real map can be plotted using this information, allowing us to draw the trajectory of the UAV throughout the simulation time.

**Figure 5:**
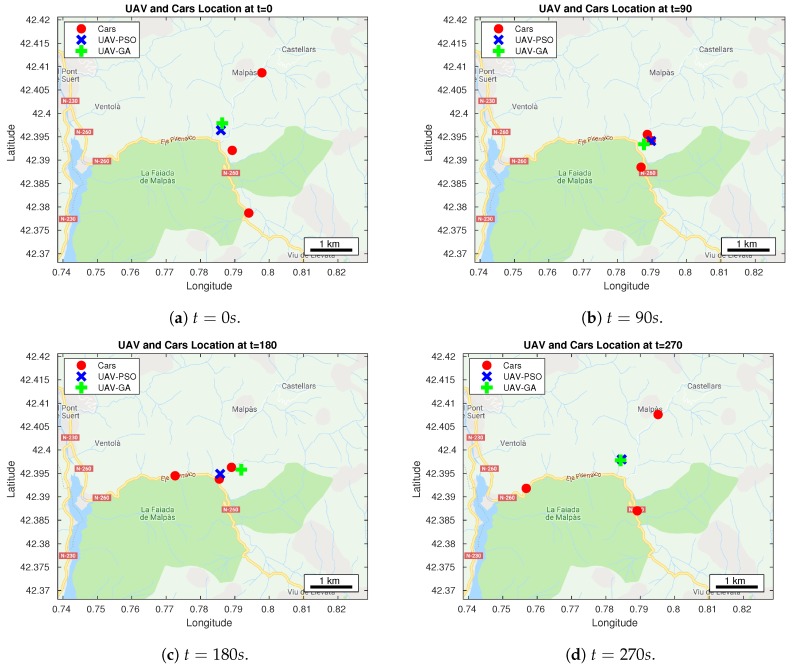
UAV and cars’ locations obtained at different timings by using both algorithms.

The locations of the UAV at specific times are represented in Figure 5 as a result from either the PSO or the GA. At t=0 s in Figure 5a, both the positions obtained using PSO and GA are located in the center of the map. Specifically, such location is at a central position with respect to the three cars on a 2D map. At t=90 s (Figure 5b), the location of the UAV obtained using PSO is near of the two cars. At this time it is not near to the other car because this car is located at a lower altitude, and hence still within LOS. On the other hand, with the GA, the UAV location is a bit more towards the other car that is far away. However, this does not make much difference as this is the time where all the cars cross with each other, and so the expected RSSI result is high. At the time where the cars move away, as depicted in Figure 5c, the positions obtained using PSO and GA are not too close from each other. In fact, the location obtained using GA is quite far away from the car moving west. On the other hand, the location obtained using PSO is closer to the center. At t=270 s, as presented in Figure 5d, the position of the UAV obtained using either PSO or GA is readjusted near to the center. This time, the cars are spreading around, and hence the UAV assumes a central position for both PSO and GA strategies.

### 5.2. UAV Trajectory Based on Positions

**Figure 6:**
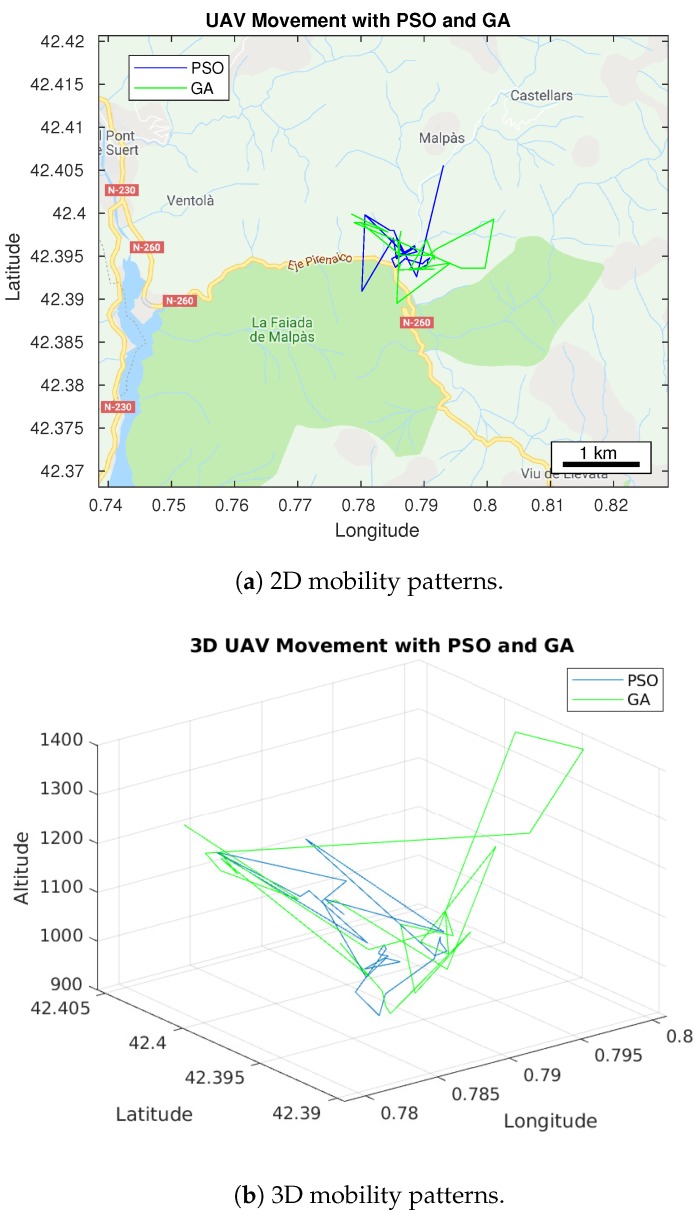
UAV trajectories generated by the PSO and GA algorithms.

The locations points obtained throughout the simulation can be sequenced with respect to time in order to get the trajectory of the UAV. This allows us to observe how the UAV should be adjusting its position in order to maintain the best connectivity towards all the cars on the ground, as shown in Figure 5. We have plotted the trajectory for both optimization algorithms in a two-dimensional and three-dimensional environment in Figure 6. These trajectories represent the most ideal path to be followed by the UAV to maintain the connection throughout the time.

The trajectory represents the optimum positions of the UAV, which change through every second, as a response to the movement of cars. Notice that the UAV should have a dynamic behavior, similarly to the mobility of the cars on the ground. As the UAV movement is exploring the 3D space, a trajectory was drawn as well, as depicted in Figure 6, where we can see the altitude variations for both sequences of UAV best positions corresponding to PSO and GA, respectively.

### 5.3. Impact on Flight Height and Altitude

**Figure 7:**
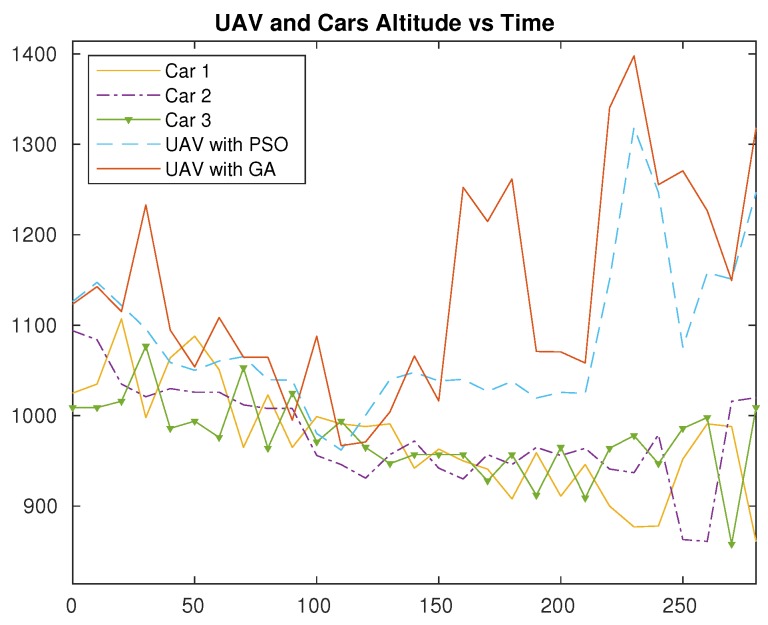
Altitude variations throughout time for the UAV and ground vehicles.

As the terrain is irregular, or hilly in the scenario, we have also obtained the altitude of the ground nodes along the time so as to have their detailed elevation information. The altitude changes as the roads have different elevation levels as they are hilly. Cars are moving at an altitude between 850 and 1100 m above sea level, as depicted in Figure 7, whereas the UAV flies at altitudes between about 970 to 1300 m above sea level if using PSO, and up to 1400 m above sea level if using GA.

Looking into Figure 7, we can observe that, at a midpoint of the simulation time, at about *t* = 120 s, the UAV altitude, as a result from both PSO and GA algorithms, is lower than the one for car 3. The UAV flies lower since the cars are located near to each other. Since the distance between ground nodes is small, the UAV does not need to fly high to maintain connectivity. However, at the end of the simulation, since the cars’ locations are far from each other, the UAV has to fly higher. So, even though the cars’ altitudes are not greater at the endpoint compared to the one at the midpoint, the UAV still has to increase its altitude since it attempts to maintain line-of-sight conditions towards all the cars. Nevertheless, notice that greater altitudes also cause the distance towards all three cars to increase. Thus, the UAV avoids flying too high to prevent losing signal quality due to higher distances, while simultaneously avoiding NLOS conditions.

The maximum altitude achieved by the UAV is at about t=230 s. It is reasonable since, at that particular time, more hills are present that obstruct the signal transmission. The altitude of car 1 is below 900 m, and this might as well result in the UAV having to fly higher to avoid NLOS towards it.

**Figure 8:**
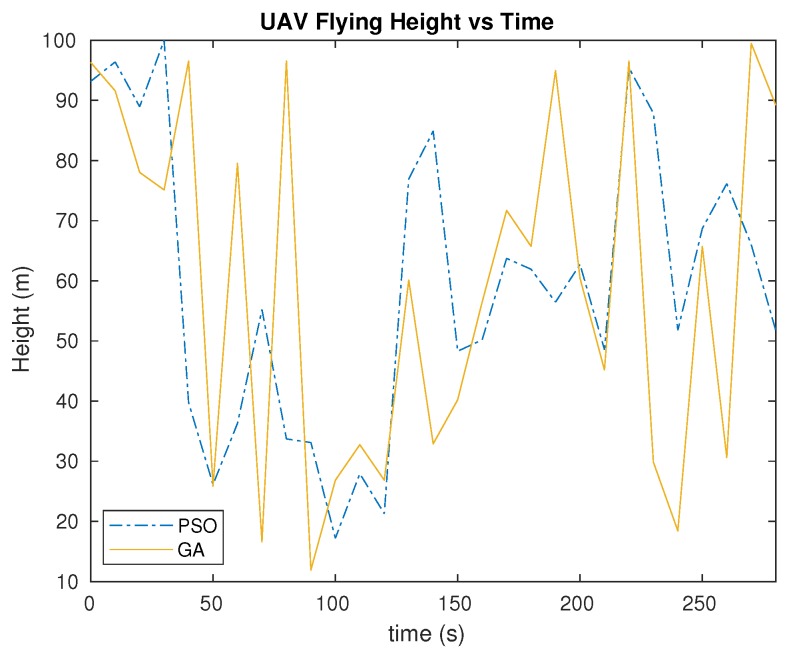
Flying height variations throughout time for the UAV.

As for comparison between PSO and GA, we can observe in Figure 8 that the trend is more or less similar, with its altitude decreasing starting from about t=40 s, and increasing at about t=130 s. Both algorithms result in a maximum altitude value at about t=230 s as well. However, the difference can be spotted in terms of consistency. With the GA approach, the curve trend is more dynamic than for the PSO approach. An example can be seen at about t=170 s. When the PSO indicates a more stable altitude change, GA shows a drastic change from an altitude of about 1250 m to 1050 m. Overall, flying height results shows that the GA trend is not as stable as the PSO trend. The heights produced from the experiment with GA have more varieties and sudden changes. An example can be seen between t=50 s and t=100 s. Within this time range, the changes are drastic for the height of the UAV, dropping from 97 m to 28 m, and then rising up again to 79 m. On the other hand, the height only rises from 28 m to 55 m with the PSO approach. In this case, if using GA, the UAV needs more effort in flying as in terms of height and altitude, it tends to be higher than when using PSO.

### 5.4. Impact on Received Signal Strength

**Figure 9:**
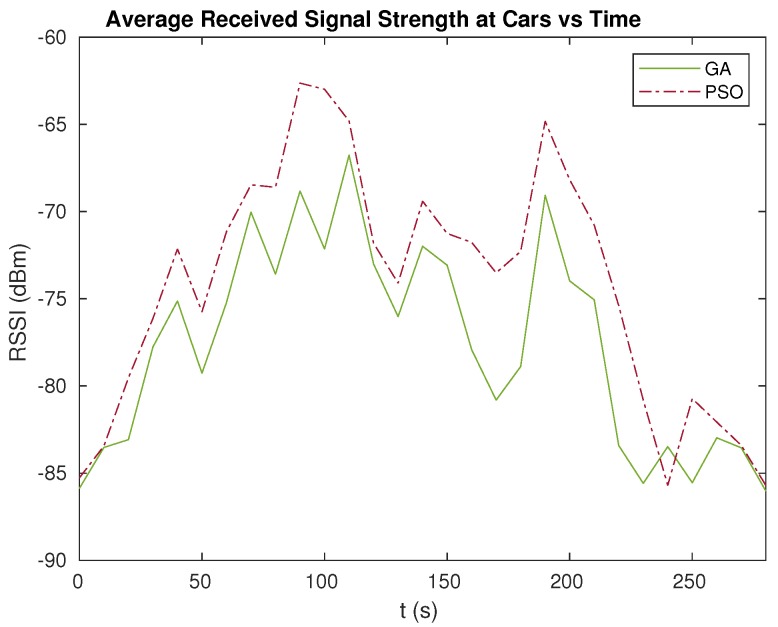
Average Received Signal Strength Indicator (RSSI) values at the receivers throughout time.

The average values of the received signal quality on the cars throughout the simulation time are represented in Figure 9 for the PSO and GA approaches. According to the figure, the lowest average values (≤85 dBm) are achieved at the beginning and end of the simulation for both GA and PSO. This is due to the fact that, at those times, the cars are spread in the area and are far from each other. Hence, the UAV is placed in-between the cars, but, since the cars are distant, the UAV had to fly higher to achieve LOS towards all three cars by adjusting its height in order to avoid blockages from the high-level terrains. This will, in turn, weaken the signal strength.

The best signal recorded in the simulation for PSO is at about t=70 s, where the RSSI is around −63 dBm. On the other hand, for GA, the best RSSI recorded is −78 dBm when the simulation time is t=130 s. At these times, the cars are located near to each other. Since the distance is smaller, the UAV does not have to fly high to achieve good transmission conditions.

**Figure 10:**
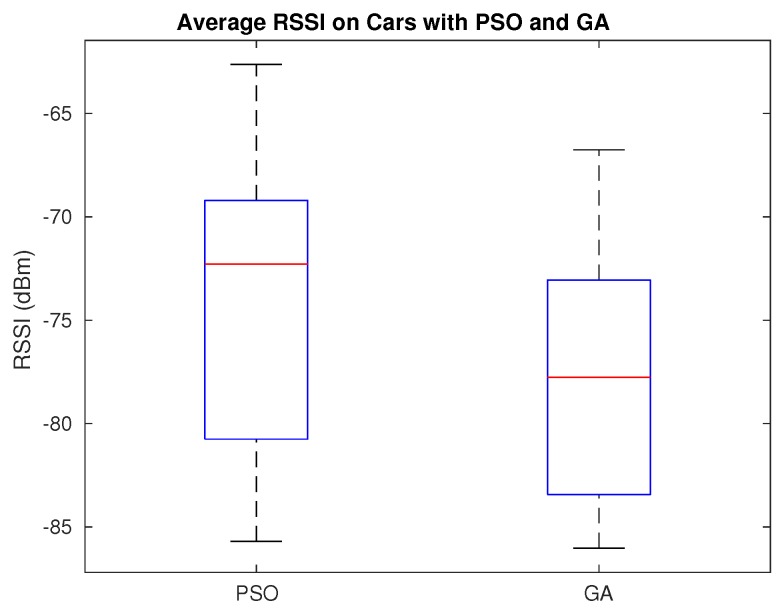
Average RSSI values at the receivers.

Figure 10 further evidences how these values are distributed, highlighting the differences between PSO and GA. From all the average RSSI values gathered, PSO shows the better results, being the majority of its values between −79 dBm and −69 dBm. On the other hand, the majority of the results for GA are between −83 dBm and −73 dBm. The mean for both algorithms also evidences the differences found: for PSO the mean result is −73 dBm, whereas for GA the mean result is −77 dBm. Overall, we clearly find that PSO is more efficient at achieving better RSSI values than GA.

### 5.5. Impact on Total Path Length

Through the sequence of locations defining the UAV trajectory, we are able to find the total length of the UAV’s path for each algorithm; these results are presented in Table 2. When using PSO as the positioning technique, the total path length is about 9.306 kilometers. On the other hand, the total path length for GA is longer, accounting to about 11.224 kilometers. The total path length using GA is longer due to the fact that not only the optimum locations obtained are sparse and quite far from each other in terms of latitude and longitude, but also, if we look at altitude variations throughout the time (see Figure 7), the position in terms of altitude changes drastically. Through these results, we can observe that the locations obtained using PSO introduce less burden to the UAV in terms of travelling distance.

### 5.6. Impact on Speed

Aside from total path length, we have also calculated how fast the UAV has to fly from one point to another for every second in the simulation. Since this kind of UAV is expected to be used for mission-specific purposes, the UAV is allowed to move at maximum speed at all times if necessary. The speed maximum values closely match the average ones as the scenario dynamics prevent it from remaining still. According to Table 2, the average speed needed for the UAV to passes through every optimum location is more than 100 km/h for both PSO and GA. To be more precise, when using PSO, the average speed needed is 119.65 km/h, which is less than the speed needed if using GA, which reaches 144.3 km/h. Again, when comparing these two optimization algorithms in terms of the average speed needed for the UAV to pass through all the optimum locations, PSO introduces lower requirements, being thus the option of choice.

## 6. Conclusions

The issue of placing UAVs to support car-to-car communications considering the restrictions of three-dimensional environments was investigated in this paper. In particular, we analyzed how different optimization algorithms can be used to find the best and optimum placement for a UAV providing support for car communications on the ground. Two types of optimization algorithms were included in our proposed placement technique: Particle Swarm Optimization, or PSO, and Genetic Algorithm, or GA. The exact sequence of UAV locations that are able to offer the best signal levels towards moving cars throughout time can be determined using PSO and GA. The quality of the signals received by the cars is the optimization parameter used to designate the location of the UAV sending the signals. By simulation, the signal quality can be calculated considering a path loss model that also counts on the elevation information for the area tested. The simulation tool can calculate the signal attenuation due to terrain blockages present based on elevation data. The positioning technique thus optimizes the position of the UAV defining its best altitude so that it can avoid terrain blockages. Based on our findings, the PSO can offer more optimized results than the GA in terms of efficiency.

To extend the work, in the future we will propose a method to find a more realistic trajectory of a UAV which takes into consideration the same parameters as in our positioning technique. The idea can be proposing a mobility model for a UAV that is aware of the dynamic movement of the cars on the ground but introducing more realistic speed requirements for the UAV.

## Figures and Tables

**Table 1:** Simulation parameters.

Parameter	Value
Transmission Power	200 mW
Antenna	5 dBi
Packet Size	1.4 kB
Message Type	BSM
Packet Sending Rate	10 Hz

**Table 2:** Path length and average flight speed for each algorithm.

Optimization Algorithm	Total Path Length (m)	Speed (km/h)
PSO	9305.90	119.65
GA	11223.69	144.30
